# Editorial: Role of epigenetic modulations and transcription factor in cardiovascular disease and coronary artery spasm: mechanisms and interventions

**DOI:** 10.3389/fcvm.2025.1546614

**Published:** 2025-02-04

**Authors:** Ioanna Koniari, Nicholas G. Kounis, Ming-Yow Hung

**Affiliations:** ^1^ Liverpool Center of Cardiovascular Science, Liverpool Heart and Chest Hospital, Liverpool, United Kingdom; ^2^ Department of Cardiology, University of Patras Medical School, Patras, Greece; ^3^ Division of Cardiology, Department of Internal Medicine, Shuang Ho Hospital, Taipei Medical University, New Taipei City, Taiwan; ^4^ Division of Cardiology, Department of Internal Medicine, School of Medicine, College of Medicine, Taipei Medical University, Taipei, Taiwan; ^5^ Taipei Heart Institute, Taipei Medical University, Taipei, Taiwan

**Keywords:** clopidogrel resistance, mitophagy, lipid metabolism-related genes, transcription factors, vascular diseases, Mendelian randomization, programmed cell death protein-1, gene set enrichment analysis

**Editorial on the Research Topic**
Role of epigenetic modulations and transcription factor in cardiovascular disease and coronary artery spasm: mechanisms and interventions

Epigenetic modifications involve silencing or downregulation/upregulation of gene expression. Dysregulation of epigenetic machinery could lead to gene expression dysregulation and cardiovascular diseases (CVDs) (1). Several epigenetic changes are documented in CVD, including (A) DNA modifications (5-methylcytosine is also used in epigenetic clocks and associated with CVD disease onset); (B) altered balance of active and repressive histone marks; (C) alterations to transcription factor binding; (D) transcriptional changes; (E) altered expression of non-coding RNAs; (F) chromatin remodeling; and (G) laminopathy and loss of heterochromatin (2).

In the paper by Giantini et al. demonstrate that one of the factors contributing to the development of post-percutaneous coronary intervention major adverse cardiovascular events (MACEs) among patients with acute coronary syndrome (ACS) is clopidogrel resistance, which may be increased by genetic and epigenetic factors, such as hypomethylation of the CYP2C19 gene and miRNA-26a upregulation. The authors found no association between clopidogrel resistance and MACEs; however, the CYP2C19∗2/∗3 loss-of function alleles genetic polymorphism could predict MACEs. Furthermore, mutant carriers of CYP2C19∗2/∗3 polymorphisms increase the risk of MACEs in 1 year. Therefore, a high proportion of clopidogrel resistance requires monitoring of platelet aggregation among post-percutaneous coronary intervention patients. The substitution of clopidogrel for ticagrelor and prasugrel in the event of hypomethylation of CYP2C19 or miRNA-26a upregulation is recommended.

In the study by using 2 Gene Expression Omnibus (GEO) datasets (GSE62646 and GSE59867) to screen the differential expression genes in peripheral blood, Yang et al. show that the transcriptional levels of 3 mitophagy-related genes, ATG5, TOMM20 and MFN2, in patients with myocardial infarction (MI) were significantly different from the control group of stable coronary artery diseases (CAD), which may hold the potential application value in the comprehensive assessment of CAD.

In the study by utilizing bioinformatics analysis methods to mine the transcriptome datasets, Wu et al. identify that Acadm, Acadvl, and Suclg1 may be the 3 key lipid metabolism-related genes (LMRGs) involved in myocardial ischemia-reperfusion injury (MI/RI). A total of 51 therapeutic agents, such as estradiol, fenofibrate, resveratrol, sulforaphane, sunitinib, bezafibrate, propylthiouracil, pravastatin, quercetin, ciprofibrate, are predicted. These identified genes or molecules could provide new recommendations on the prevention and management of MI/RI.

In the paper by Hu et al. provide a narrative review of a widely recognized transcription factor (TF), krüppel-like family (KLF), in epigenetically regulating vascular endothelial cells (VECs) and vascular smooth muscle cells (VSMCs). KLF11 inhibits VSMCs apoptosis and enhances their contractile function. KLF4 attenuates the formation of VSMCs-derived foam cells, reduces endothelial dysfunction, activates endothelial nitric oxide synthase and vasodilatation, and inhibits endothelin-1-induced vasoconstriction. While the understanding of TFs is still limited, the integration of the prior proposed TF activity sequencing technology will allow us to dissect the molecular details in the regulation of cell fate of VECs and VSMCs.

In the paper by Zeng et al. conduct a mendelian randomization (MR) analysis to investigate the causal relationship between programmed cell death protein-1 (PD-1), its ligand PD-L1 and CAD, complemented by gene set enrichment analysis (GSEA) for further validation. Inverse-variance weight (IVW) analysis showed a causal association between PD-1, PD-L1 and chronic CAD. This study provided evidence of a bidirectional causal relationship between PD-1 and chronic CAD and a protective association between chronic CAD and PD-L1. Gene set related to PD-1/PD-L1 was revealed downregulated in CHD by GSEA, which consolidate the MR result. Thus, PD-1/PD-L1 is a new potential independently inflammatory diagnostic biomarker for CAD and merits further study as a potential diagnostic target.

By understanding the mechanism of treatment failure and by advancing the diagnosis of CVD, including coronary artery spasm (CAS), a new stage of individualized therapy may arise with assessments of epigenetic modulations and transcription factors in the same way that lipid profile, blood sugar, kidney and liver function are measured, thus improving the healthcare of patients with CVD and CAS. These results provided by the aforementioned 5 studies will motivate new basic and clinical research to shed light on the fundamental pathological mechanisms of CVD and CAS, and, hopefully, will identify potential new candidate targets for therapeutic intervention because our current available armamentarium for CVD and CAS is extremely limited.

## References

[1] Soler-Botija C Gálvez-Montón C Bayés-Genís A. Epigenetic biomarkers in cardiovascular diseases. Front Genet. (2019) 10:950. 10.3389/fgene.2019.00950 31649728 PMC6795132

[2] Herman AB Occean JR Sen P. Epigenetic dysregulation in cardiovascular aging and disease. J Cardiovasc Aging. (2021) 1:10. 10.20517/jca.2021.16 34790973 PMC8594871

